# Health Care Providers’ Performance, Mindset, and Attitudes Toward a Neonatal Resuscitation Computer-Based Simulator: Empirical Study

**DOI:** 10.2196/21855

**Published:** 2020-12-21

**Authors:** Maria Cutumisu, Simran K Ghoman, Chang Lu, Siddhi D Patel, Catalina Garcia-Hidalgo, Caroline Fray, Matthew R G Brown, Russell Greiner, Georg M Schmölzer

**Affiliations:** 1 Department of Educational Psychology Faculty of Education University of Alberta Edmonton, AB Canada; 2 Centre for the Studies of Asphyxia and Resuscitation Neonatal Research Unit Royal Alexandra Hospital Edmonton, AB Canada; 3 Department of Computing Science Faculty of Science University of Alberta Edmonton, AB Canada; 4 Department of Pediatrics Faculty of Medicine and Dentistry University of Alberta Edmonton, AB Canada

**Keywords:** infant, newborn, delivery room, neonatal resuscitation, performance, neonatal resuscitation program, serious games, computer-based game simulation, mindset

## Abstract

**Background:**

Neonatal resuscitation involves a complex sequence of actions to establish an infant’s cardiorespiratory function at birth. Many of these responses, which identify the best action sequence in each situation, are taught as part of the recurrent Neonatal Resuscitation Program training, but they have a low incidence in practice, which leaves health care providers (HCPs) less prepared to respond appropriately and efficiently when they do occur. Computer-based simulators are increasingly used to complement traditional training in medical education, especially in the COVID-19 pandemic era of mass transition to digital education. However, it is not known how learners’ attitudes toward computer-based learning and assessment environments influence their performance.

**Objective:**

This study explores the relation between HCPs’ attitudes toward a computer-based simulator and their performance in the computer-based simulator, RETAIN (REsuscitation TrAINing), to uncover the predictors of performance in computer-based simulation environments for neonatal resuscitation.

**Methods:**

Participants were 50 neonatal HCPs (45 females, 4 males, 1 not reported; 16 respiratory therapists, 33 registered nurses and nurse practitioners, and 1 physician) affiliated with a large university hospital. Participants completed a demographic presurvey before playing the game and an attitudinal postsurvey after completing the RETAIN game. Participants’ survey responses were collected to measure attitudes toward the computer-based simulator, among other factors. Knowledge on neonatal resuscitation was assessed in each round of the game through increasingly difficult neonatal resuscitation scenarios. This study investigated the moderating role of mindset on the association between the perceived benefits of understanding the terminology used in the computer-based simulator, RETAIN, and their performance on the neonatal resuscitation tasks covered by RETAIN.

**Results:**

The results revealed that mindset moderated the relation between participants’ perceived terminology used in RETAIN and their actual performance in the game (*F*_3,44_=4.56, R^2^=0.24, adjusted R^2^=0.19; *P*=.007; estimate=–1.19, SE=0.38, t_44_=–3.12, 95% CI –1.96 to –0.42; *P*=.003). Specifically, participants who perceived the terminology useful also performed better but only when endorsing more of a growth mindset; they also performed worse when endorsing more of a fixed mindset. Most participants reported that they enjoyed playing the game. The more the HCPs agreed that the terminology in the tutorial and in the game was accessible, the better they performed in the game, but only when they reported endorsing a growth mindset exceeding the average mindset of all the participants (*F*_3,44_=6.31, R^2^=0.30, adjusted R^2^=0.25; *P*=.001; estimate=–1.21, SE=0.38, t_44_=−3.16, 95% CI –1.99 to –0.44; *P*=.003).

**Conclusions:**

Mindset moderates the strength of the relationship between HCPs’ perception of the role that the terminology employed in a game simulator has on their performance and their actual performance in a computer-based simulator designed for neonatal resuscitation training. Implications of this research include the design and development of interactive learning environments that can support HCPs in performing better on neonatal resuscitation tasks.

## Introduction

### Background

Approximately 1 in 10 infants worldwide will require some degree of neonatal resuscitation at birth to support their circulation and breathing [1]. Infants may receive assistance across 4 categories of sequential actions: initial stabilization (provide warmth, clear airways, dry, stimulate, and reevaluate), ventilation, chest compressions, and administration of epinephrine and volume expansion [2]. These actions, including a continuous evaluation of the infant, must be performed rapidly, yet accurately. Moreover, these actions are usually performed collaboratively in teams of specialized health care providers (HCPs), which adds complexity to an already high-stress, time-sensitive task of saving an infant’s life, as miscommunication can lead to errors and waste precious time [3]. Furthermore, 1% of the infants require more extensive resuscitation measures, such as chest compression and epinephrine [4].

Many HCPs may encounter these types of high-acuity low-opportunity events once in their careers. Such events require exceptional expertise, team dynamics, as well as cognitive and psychomotor acuity. Due to the rare occurrence of these highly specialized events and their collaborative, team-based nature, breakdown in HCP communication is the leading cause of neonatal death [5]. In such situations, deviation from the neonatal resuscitation protocol can occur. Specifically, it is estimated that human error causes over two-thirds of morbidity and mortality in neonatal resuscitation cases [5]. Moreover, these errors are directly proportional to the complexity of the resuscitation [6].

HCPs experience decline in skills after training over time [7]. This presents a challenge, given the limited exposure of HCPs (novice and expert alike) to challenging, realistic, complex, high-risk, and rarely occurring scenarios, compounded by infrequent hands-on experiences in traditional medical education. One potential strategy to compensate for this scarcity is periodic simulation-based training to acquire and maintain expertise [8], especially in medical education [9]. Health care education has employed simulations that represent abstractions of real-life scenarios and that incorporate expert knowledge models to provide alternative opportunities for training and learning [10]. Moreover, neonatal resuscitation guidelines recommend the use of simulation-based medical education as a solution to mitigate the loss of skills over time and to decrease human error in the delivery room [11]. Several researchers have used computer-based simulations successfully in pediatric and neonatal resuscitation for the last 2 decades to improve performance and learning [12-16].

Simulations have several benefits over more traditional training experiences. They offer deliberate practice and experiential learning opportunities associated with better learning, especially when they foster a safe and supportive environment, where making mistakes is welcomed as an opportunity to uncover and remedy gaps in knowledge [17,18]. Simulation is a safer alternative, as no real patients are required. Furthermore, it puts neither the patient nor the trainee at risk, especially in neonatal resuscitation when the outcome in the real situation may be the loss of life or in simulating infectious diseases when the outcome may be the contamination of the trainee. In neonatal resuscitation, simulations showed several benefits for HCPs [19-21]. In some cases, simulations can be used anytime and anywhere and can reproduce rarely occurring training scenarios and can tune the difficulty and complexity of the scenario to exemplify the phenomena of interest. Simulations can also provide immediate expert feedback and diagnostic assessment, which were also found to support learning. Importantly, the experiential nature of the simulations and the similarity of simulated scenarios with real-life situations may help the participant transfer the skills learned, for instance, from the simulation to the delivery room [22]. However, very few studies were conducted to specifically target transfer. For instance, in a recent scoping review of medical student training in eHealth, none of the articles reviewed aimed to demonstrate that the eHealth training of medical students transferred outside the simulation environment [23].

### The REsuscitation TrAINing Simulator

The RETAIN (REsuscitation TrAINing) simulator employed in this study is a computer role-playing game [24,25] that was designed to support novice HCPs in acquiring neonatal resuscitation knowledge and to assist expert HCPs in refreshing their knowledge, especially by exposing them to novel, complex, and rarely occurring scenarios, in between taking the Neonatal Resuscitation Program (NRP) refresher courses [26]. The game is also relatively short, up to 10 minutes, which fits in an HCP’s busy schedule, and it is easily accessible, as it only requires a computer.

Although there is a paucity of computer-based simulators, they have been found to improve knowledge and decision-making skills [27-30]. However, few studies have examined participants’ experiences in these environments and the attitudes (eg, mindsets or theories of intelligence) they bring to these tasks [31]. Thus, this study adds to the research body on neonatal resuscitation simulators by analyzing the survey responses and computer-based game simulator performance of HCPs to gain an insight into their perceptions of the simulation environment, their performance on these tasks, and the relationship between their attitudes and performance. This exploration is prompted by the belief that individuals who endorse a growth mindset are more interested in mastery and work harder to overcome barriers and achieve their goals, as they believe that intelligence and ability are malleable; concomitantly, those who endorse a fixed mindset are more interested in performance and do not work as hard, as they do not believe that they can change their abilities [31]. The objectives of this study were (1) to determine whether attitudes toward the simulator hinder or enhance HCPs’ performance in a neonatal resuscitation computer-based game simulation and (2) to examine whether the HCP’s mindset moderates this relationship between attitudes and performance. We hypothesized that HCPs’ mindset would strengthen the relation between their perceived performance, given their understanding of the terminology used in the neonatal resuscitation simulator and their actual performance in this environment.

This study contributes to understanding the influence of HCPs’ mindset and perceptions of computer-based game simulations for neonatal resuscitation on their performance. Moreover, these noncognitive factors are examined in conjunction with HCPs’ performance in increasingly difficult neonatal resuscitation scenarios, providing an insight into cognitive factors and into the impact of attitudes on performance. Lessons learned from this study may help medical education instructors incorporate computer-based game simulations in their training and instructional practice.

## Methods

### Participants

Fifty HCPs (45 females, 4 males, and 1 not reported), who had completed their NRP [26] training within the 24 months preceding this study, were recruited voluntarily from the neonatal intensive care unit (NICU) at the Royal Alexandra Hospital, Edmonton, Canada, a tertiary perinatal center delivering over 6500 infants every year. The sample consisted of 16 respiratory therapists, 33 registered nurses and nurse practitioners, and 1 physician, which was representative of the HCP population within the NICU. The study was performed at the bedside in the NICU and it was approved by the Human Research Ethics Board at the University of Alberta (Pro00064234). Written informed consent was obtained from all HCPs prior to participation and no participants were excluded. None of the participants had any prior experience with the RETAIN computer-game simulation.

### Study Setup

The study was conducted based on a computer-based game simulator RETAIN (for HCPs) at the simulation laboratory at the Centre for the Studies of Asphyxia and Resuscitation [32]. RETAIN was designed to support HCPs in practicing their neonatal resuscitation knowledge in between regular NRP [26] refresher courses. In the RETAIN computer-based game simulator, participants, assuming the role of a neonatologist, tackled increasingly difficult simulated neonatal resuscitation scenarios in each of the 3 rounds of the game, each of which required first repeating and then extending the steps taken on the previous rounds. Their avatar (ie, a medical doctor) needed to perform 4 categories of sequential actions: initial stabilization, ventilation, chest compressions, and administration of epinephrine and volume expansion to assist an infant at birth. For example, the last game round required the player to perform mask ventilation, chest compression, and administer epinephrine to stabilize the infant. The participants had a limited amount of time to complete the neonatal resuscitation scenario presented in each game round, as the game provided a countdown that simulated the urgency of a real-world, high-stakes, fast-paced delivery room. Players were allowed to advance to the next game round only if they made at most 4 mistakes in the current game round. Otherwise, they were required to repeat that round.

### Procedure and Data Collection

All participants completed 2 surveys—1 before and 1 after completing the computer-based simulation. The presurvey included demographic and educational background items (eg, time in months since the participant’s last NRP course), whereas the postsurvey consisted of attitudinal items, including their views on the current computer simulation and mindset (eg, the terminology or phrasing used did not impede your ability to complete the steps). Attitudinal items included in the postsurvey used a 5-point Likert scale (1: Strongly disagree, 2: Disagree, 3: Neutral, 4: Agree, and 5: Strongly agree).

Between the surveys, the participants played the RETAIN computer game. This particular game version was implemented using the Aurora game engine of the award-winning *Neverwinter Nights* computer role-playing game [33]. The game started with a short tutorial presenting a practice scenario that familiarized players with the mechanics of the game. Then, the participants played 3 consecutive game rounds that presented resuscitation scenarios of increasing difficulty, each encompassing the steps taken in the previous rounds. The game took an average of 8.47 minutes to play. More details about this game are presented in previous studies [24,25,34]. Learning analytics regarding participants’ performance and behaviors were tracked within the computer-game simulator.

### Performance Measures

Number of Tries: The outcome variable employed in this study represents the number of tries performed in the game. This measure ranged from 32 (ie, the participant solved all the scenarios from the first try) to 54 (ie, the participant took more tries to solve the game scenarios).

### Attitudinal Measures

All the following items were rated on a Likert scale from 1 (*Strongly disagree*) to 5 (*Strongly agree*), as mentioned above.

1. Enjoyment: This variable captured the participants’ response values to the following statement, that is, “Did you enjoy playing this game?”
2. Tutorial Terminology: The predictor variable captured the participants’ response values to the following statement, that is, “The terminology used did not impede your ability to complete the steps.”
3. Terminology Used: We also considered another predictor variable that captured the participants’ response values to the following statement about the entire game, that is, “The terminology or phrasing used did not impede your ability to complete the steps.”
4. Mindset: The moderator variable captured the participants’ response values to 4 items that probed participants’ theories of intelligence (ie, mindsets) [31]. Two items were related to fixed mindset or the belief that intelligence is fixed (Fixed Mindset 1: “You can’t really do much to change how good you are at your job” and Fixed Mindset 2: “You can learn new things, but you cannot really change how good you are at your job”), while 2 other items were related to growth mindset or the belief that intelligence is malleable (Growth Mindset 1: “You can always change how good you are at your job” and Growth Mindset 2: “You can get better at your job with practice”). All items were positively stated, except for the 2 fixed-mindset items, which were reverse-coded. Then, a Mindset variable was obtained by adding the growth-mindset items and the reversed fixed-mindset items: 12 – (Fixed Mindset 1 + Fixed Mindset 2) + (Growth Mindset 1 + Growth Mindset 2).

### Statistical Analyses

All analyses were performed using version 4.0.2 of the R [35] statistical software. They included descriptive statistics and tests of association, including correlation and regression analyses, to test the moderation effect for continuous interactions.

Descriptive analyses: First, we computed the summary statistics of the study variables by using the *summary* function and the *describe* function of the *Hmisc* [36] package in R. Second, we explored the relationships between the variables included in this study. Specifically, we conducted two-fold correlation analyses between the outcome variable and the 2 mean-centered predictor and moderator variables: (1) Spearman correlations using the *corr.test* function of the *psych* [37] package, which also generates confidence intervals, and (2) robust correlations to compute the percentage-bend correlation coefficient, using the *pbcor* function of the *WRS2* [38] package in R.

Multiple regression analyses: These analyses, which were conducted using the *lm* built-in function in R [35], probed whether the moderator influenced the strength of the relation between each predictor variable and the outcome variables employed in this study. First, linearity assumptions of the multiple linear regression analysis were tested using the *gvlma* [39] package in R. Multicollinearity tests were conducted to ascertain whether multicollinearity was problematic for any of the models. The updated quantile-comparison plots for the robust models are shown in Figures S2, S3, S5, and S6 in Multimedia Appendix 1. Then, the Johnson-Neyman [40,41] technique was conducted to test the interaction, as the variables involved were continuous. We used the *sim_slopes* and the *johnson_neyman* functions of the *interactions* [42] (former *jtools*) R package to determine the *regions of significance* (ie, the precise range of the moderator values for which the main effect of the predictor on the outcome is statistically significantly different from zero) for simple slopes. The Johnson-Neyman technique displays 95% confidence bands around the regression line (ie, representing a multiplicative interaction effect model) showing how the main effect varies across the values of a moderator. Both the predictor and the moderator variables were mean-centered for this analysis to account for the multicollinearity caused by the association between the independent variables and for ease of result interpretation.

## Results

### Descriptive Analyses

Table 1 presents the descriptive statistics together with the correlation results for both Spearman and robust correlations.

**Table 1:** Descriptive statistics for the study variables, Spearman correlation coefficients and their confidence intervals for the study variables, and robust correlation coefficients.

Variable		Tutorial Terminology	Terminology Used	Mindset	Number of Tries
**Spearman correlations**					
	Tutorial Terminology	—^a^			
	Terminology Used	0.75^b^ [0.60, 0.85]	—		
	Mindset	0.20 [–0.08, 0.45]	0.12 [–0.17, 0.38]	—	
	Number of Tries	0.08 [–0.20, 0.35]	0.09 [–0.20, 0.36]	–0.15 [−0.41, 0.14]	—
**Robust correlations**					
	Tutorial Terminology	—			
	Terminology Used	0.73^b^	—		
	Mindset	0.23	0.14	—	
	Number of Tries	0.11	0.09	–0.15	—
Participants (n)		50	50	48	50
Mean (SD)		4.46 (0.61)	4.30 (0.71)	17.79 (2.32)	35.72 (4.16)
Minimum		2	2	12	32
Maximum		5	5	20	54
Skewness		–1.15	–1.15	–0.86	2.16
Kurtosis		2.64	2.11	–0.01	5.69
SE		0.09	0.10	0.34	0.59

^a^Not applicable.

^b^The correlation was significant at *P*<.001.

**Figure 1:**
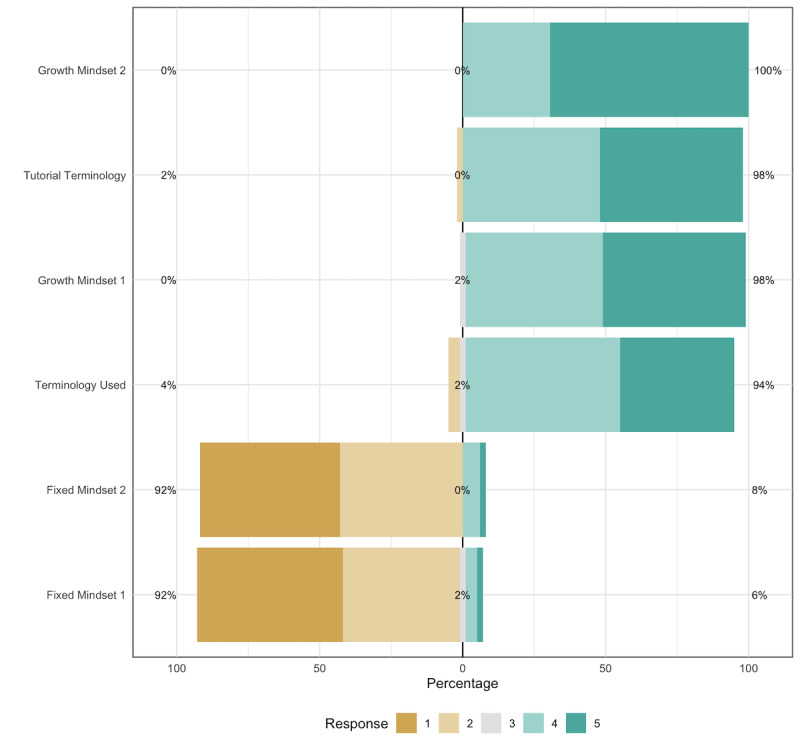
Responses on a 5-point Likert scale to the items included in this study. 1: Strongly disagree; 2: Disagree; 3: Neutral; 4: Agree; and 5: Strongly agree.

Figure 1 shows that most participants agreed or strongly agreed with the statements regarding *Tutorial Terminology* and *Growth Mindset* but disagreed and strongly disagreed with the statements related to *Fixed Mindset*. Additionally, most participants reported that they enjoyed the game, with a mean (SD) of 4.04 (0.68) as well as a median and a mode of 4 on a self-reported item measuring participants' gameplay enjoyment.

### Multiple Regression Analyses

We conducted a moderation analysis to examine whether the relationship between the continuous predictor, *Tutorial Terminology* in Model 1 (*Terminology Used* in Model 2, respectively) and the dependent continuous variable, *Number of Tries*, was moderated by the continuous variable, *Mindset*.

Multicollinearity tests yielded variance inflation factor (VIF) values near 1.0 and less than 5.0 for both Model 1 (VIFpredictor=1.14, VIFmoderator=1.01, and VIFinteraction=1.13) and Model 2 (VIFpredictor=1.29, VIFmoderator=1.00, and VIFinteraction=1.29), indicating that multicollinearity was not problematic for any of the 2 models.

The Shapiro-Wilk normality test revealed that the outcome variable was not normally distributed (W=0.75, *P*<.001). The residuals of Model 1 and Model 2 were also not normally distributed, as shown in the quantile-comparison plot of Figure S1 and Figure S4, respectively, included in Multimedia Appendix 1. Thus, a robust linear regression, which also provides robustness to outliers, was conducted using the *lmrob* function of the *robustbase* [43] packages in R for heteroscedasticity-robust fitting of linear regression models to compute a fast M estimator [44,45].

#### Model 1: Tutorial Terminology, Mindset, and Number of Tries

The findings shown in Table 2 revealed that the more the HCPs agreed that the *Tutorial Terminology* did not impede their ability to complete the tutorial scenarios, the fewer tries they needed to complete the game, but this association was significant only for those who endorsed more of a growth mindset (ie, exceeded the average mindset value across the sample).

**Table 2:** Johnson-Neyman moderator analysis for model 1: Mindset moderates the relationship between Tutorial Terminology and Number of Tries across the game^a^.

Effect (n=48)			Estimate	SE	*t (df)*	95% CI	*P* value
**Coefficients**							
	Intercept		35.67	0.50	70.91 (44)	[34.65, 36.68]	*<.001* ^b^
	Tutorial Terminology		–1.36	0.87	–1.55 (44)	[–3.12, 0.40]	.13
	Mindset		–0.16	0.22	–0.75 (44)	[–0.60, 0.28]	.46
	Tutorial Terminology:Mindset		–1.21	0.38	–3.16 (44)	[–1.99, –0.44]	.003
**Simple slopes analysis**							
	**When moderator^c^ = –2.32 (-1SD)**						
		Slope of moderator	1.46	1.44	1.01 (44)	N/A^d^	.32
		Conditional intercept	36.01	0.71	50.56 (44)	N/A	*<.001*
	**When moderator = 0.00 (Mean)**						
		Slope of moderator	–1.36	0.87	–1.55 (44)	N/A	.13
		Conditional intercept	35.70	0.50	71.11 (44)	N/A	*<.001*
	**When moderator = 2.32 (+1SD)**						
		Slope of moderator	–4.18	1.02	–4.11 (44)	N/A	*<.001*
		Conditional intercept	35.38	0.72	49.35 (44)	N/A	*<.001*
**Robust linear regression**							
	Intercept		34.50	0.39	88.09 (44)	[33.72, 35.29]	*<.001*
	Tutorial Terminology		1.31	0.68	1.92 (44)	[–0.07, 2.69]	.06
	Mindset		–0.13	0.13	–0.96 (44)	[–0.39, 0.14]	.34
	Tutorial Terminology:Mindset		–0.72	0.27	–2.70 (44)	[–1.26, –0.18]	.01

^a^Model fit: *F*_3,44_=6.31, R^2^=0.30, adjusted R^2^=0.25; *P*=.001.

^b^The values in italics were significant at *P*<.001.

^c^The moderator was the centered mindset variable.

^d^N/A: not applicable.

The Johnson-Neyman interval indicated that when *Mindset* was outside the interval [–5.10, 0.28], the slope of *Tutorial Terminology* was statistically significant at the *P*<.05 level, with the range of observed values of *Mindset* being [–5.79, 2.21]. Specifically, this relationship was significant when the value of the mean-centered *Mindset* variable was included in the intervals [–5.79, –5.10) or (0.28, 2.21], which corresponds to the value of the noncentered *Mindset* variable being included in the intervals [12, 12.69) or (18.07, 20], as its mean was 17.79 as shown in Table 1. The linear model fitted using an MM-type estimator (ie, the M estimator) yielded the same results as the original nonrobust linear regression model and it identified 4 outlier observations, as shown in Table 2.

**Figure 2:**
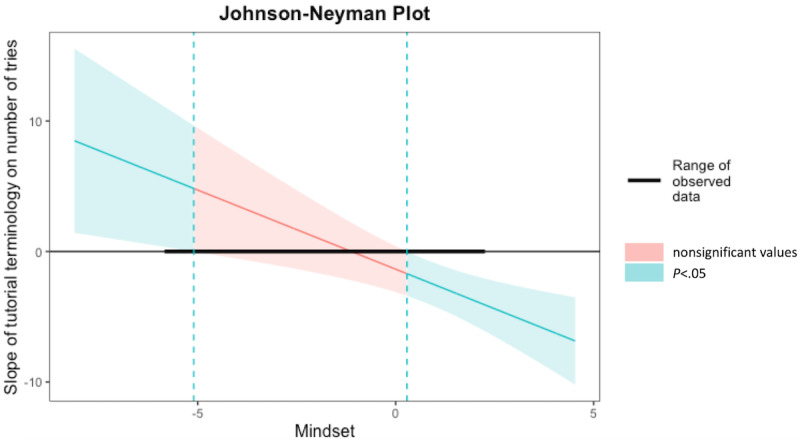
The Johnson-Neyman interaction intervals for Model 1 with Tutorial Terminology as a predictor. The region of significance is determined by the locations where the upper and lower bounds of the 95% confidence interval intersect zero.

Figure 2 displays a plot of conditional effects showing how the effect of the influence exerted by the independent variable (*Tutorial Terminology*) on the dependent variable (*Number of Tries*) is conditional on the full range of the moderator (*Mindset*). This figure displays the adjusted effect of the *Tutorial Terminology* on *Number of Tries* (y axis) across all continuous values of *Mindset* (x axis). For any values of the moderator for which the confidence bands do not contain 0, the effect of the predictor on the outcome is significantly different from 0 at the *P*=.05 level.

**Figure 3:**
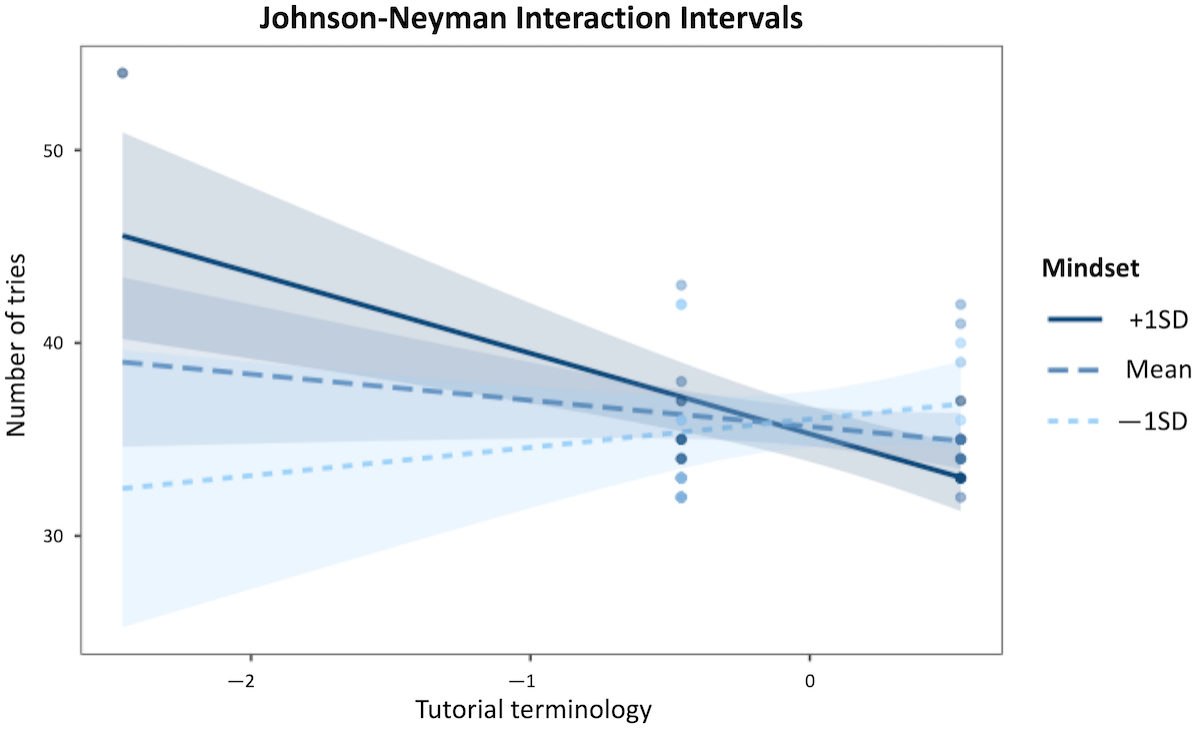
Model 1 interaction plot: the relationship between the Tutorial Terminology predictor and the Number of Tries dependent variable is significant only for very low or very high levels of the Mindset moderator.

Figure 3 shows the effect of *Tutorial Terminology* (x axis) on the *Number of Tries* (y axis) at 3 levels of *Mindset*: low (1 standard deviation lower than the mean), moderate (mean), and high (1 standard deviation higher than the mean).

#### Model 2: Terminology Used, Mindset, and Number of Tries

The findings shown in Table 3 also revealed that the more the HCPs agreed that the overall *Terminology Used* in the game did not impede their ability to complete the steps, the fewer tries they took to complete the game, but this association was significant only for those who endorsed more of a growth mindset (ie, exceeded the average mindset value).

**Table 3:** Johnson-Neyman moderator analysis for model 2: Mindset moderates the relationship between Terminology Used and Number of Tries across the game^a^.

Effect (n=48)			Estimate	SE	*t (df)*	95% CI	*P* value
**Coefficients**							
	Intercept		35.56	0.52	68.05 (44)	[34.51, 36.61]	*<.001* ^b^
	Terminology Used		–0.01	0.84	–1.01 (44)	[–1.70, 1.69]	.99
	Mindset		–0.21	0.23	–0.92 (44)	[–0.67, 0.25]	.36
	Terminology Used:Mindset		–1.19	0.38	–3.12 (44)	[–1.96, –0.42]	.003
**Simple slopes analysis**							
	**When moderator^c^ = –2.32 (–1SD)**						
		Slope of moderator	2.77	1.48	1.86 (44)	N/A^d^	.07
		Conditional intercept	36.02	0.74	48.45 (44)	N/A	*<.001*
	**When moderator = 0.00 (Mean)**						
		Slope of moderator	–0.01	0.84	–0.01 (44)	N/A	.99
		Conditional intercept	35.56	0.52	68.06 (44)	N/A	*<.001*
	**When moderator = 2.32 (+1SD)**						
		Slope of moderator	–2.78	0.89	–3.13 (44)	N/A	*<.001*
		Conditional intercept	35.10	0.74	47.21 (44)	N/A	*<.001*
**Robust linear regression**							
	Intercept		34.72	0.53	66.12 (44)	[33.66, 35.78]	*<.001*
	Terminology Used		1.40	0.72	1.95 (44)	[–0.04, 2.84]	.06
	Mindset		–0.28	0.18	–1.60 (44)	[–0.64, 0.07]	.12
	Terminology Used:Mindset		–0.66	0.29	–2.30 (44)	[–1.23, –0.08]	*.03*

^a^Model fit: *F*_3,44_=4.56, R²=0.24, adjusted R²=0.19; *P*=.007.

^b^The values in italics were significant at *P*<.001.

^c^The moderator was the centered mindset variable.

^d^N/A: not applicable.

**Figure 4:**
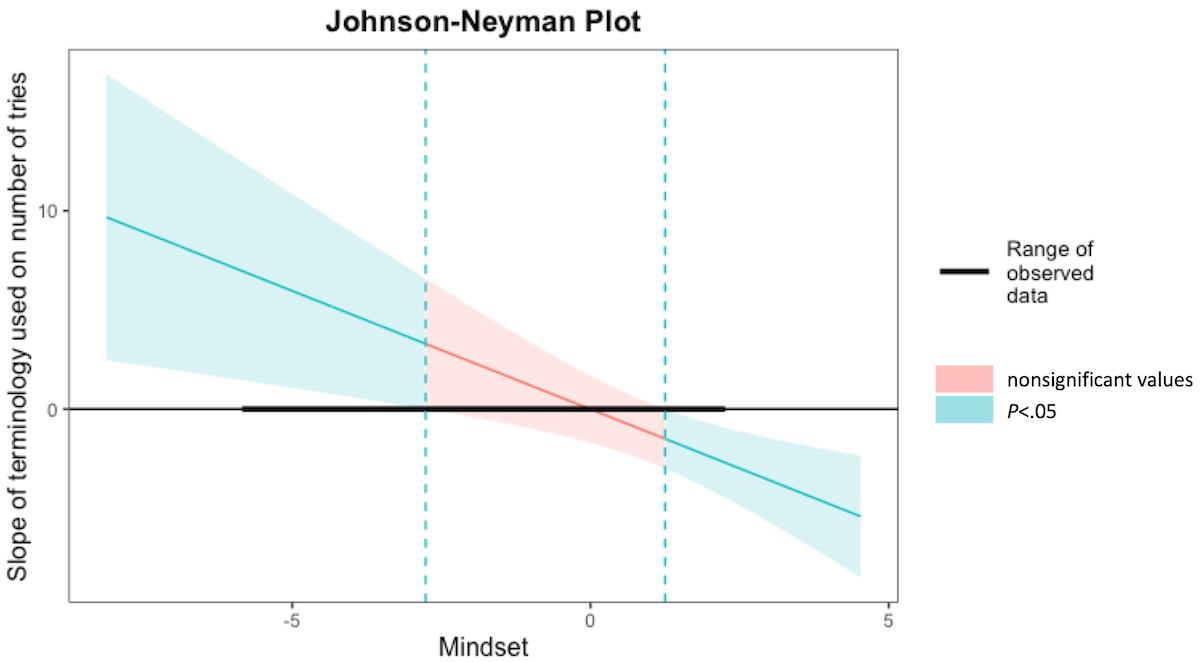
The Johnson-Neyman interaction intervals for Model 2 with Terminology Used as a predictor. The narrow area of the confidence bands represents smaller errors of estimate.

**Figure 5:**
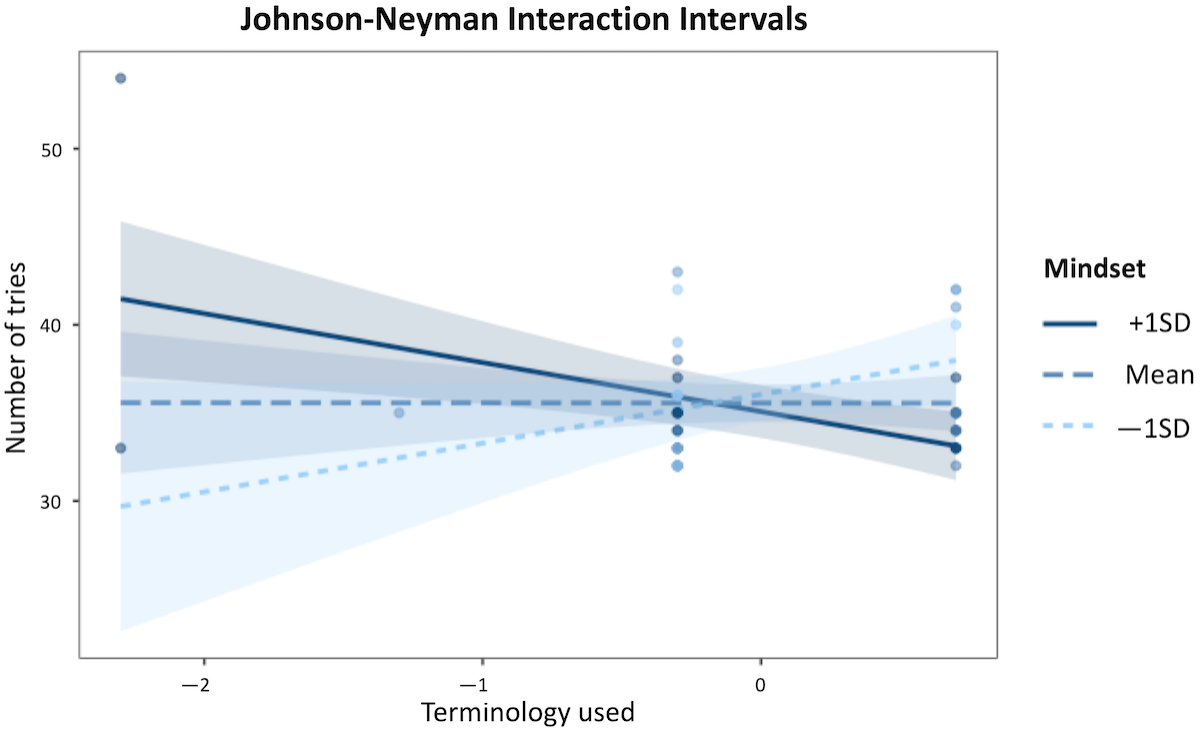
Model 2 interaction plot: the relationship between the Terminology Used predictor and the Number of Tries dependent variable is significant only for very low or very high levels of the Mindset moderator.

The Johnson-Neyman interval yielded that when *Mindset* was outside the interval [–2.76, 1.25], the slope of *Terminology Used* was statistically significant at the *P*<.05 level, with the range of observed values of *Mindset* being [–5.79, 2.21], as in Model 1. Specifically, this relationship was significant when the value of the mean-centered *Mindset* variable was included in the intervals [–5.79, –2.76) or (1.25, 2.21], which is equivalent to the value of the noncentered *Mindset* variable being included in the intervals [12, 15.03) or (19.04, 20], as its mean was 17.79 as shown in Table 1. As before, the linear model fitted using an MM-estimator yielded the same results as the original nonrobust linear regression model and it identified 1 outlier, as shown in Table 3. Figure 4 shows a plot of conditional effects for Model 2. Figure 5 shows the effect of *Terminology Used* (x axis) on *Number of Tries* (y axis) for 3 levels of *Mindset*.

## Discussion

### Primary Outcomes

The results of this study revealed that the more the HCPs agreed that the terminology used in both the tutorial and the game did not impede their game experience, the better they performed in the game, but this was only when they endorsed growth-mindset levels exceeding the average growth mindset. Although there have been recent advances in computer-based simulations in terms of their high fidelity and realistic portrayal of real-world environments, few studies have explored HCPs’ attitudes toward computer-based simulators, and even fewer have linked their attitudes to mindset and performance. The findings of this study reveal that most participants agreed or strongly agreed that the terminology used in the tutorial and in the game did not impede their ability to successfully complete the game. Moreover, the results show that participants strongly endorsed growth mindset and weakly endorsed fixed mindset. Further, the findings show that HCPs largely enjoyed playing the game, which was also echoed in other computer-based simulations for neonatal resuscitation [46,47]. Together with the results regarding the use of terminology in the game, the findings suggest that HCPs appreciate the guided apprenticeship and realism of the simulator. This result is echoed in the literature, with several studies in anesthesiology showing that the use of simulators enhanced participants’ understanding [48].

The results of this study also show that the more the participants agreed with statements about the terminology used in both the tutorial and in the game, the fewer tries they needed to complete the game (ie, the better they performed in the game) but only when they endorsed more of a growth mindset. This is represented by the right region of significance in the Johnson-Neyman interaction plots and by the negative slope when mindset is around 1 standard deviation above the mean. Conversely, the more the participants agreed with statements about the terminology used in both the tutorial and in the game, the more tries they made in the game (ie, the worse they performed in the game) but only when they endorsed more of a fixed mindset. This is represented by the left region of significance in the Johnson-Neyman interaction plots and by the positive slope when mindset is around 1 standard deviation below the mean. A previous research study found that endorsing a growth mindset moderated the relationship between the time elapsed from the last NRP refresher course and the number of mistakes in a neonatal resuscitation task performed in a video game [25], thereby showing the mindset is an important factor to consider in relation to performance. However, that study used 2 constructs for mindset (1 for growth and 1 for fixed mindset), in contrast to 1 mindset continuum employed in this study. Moreover, recent research studies suggest that mindset may influence performance differentially if it is generic or domain-specific [49].

Computer-based game simulators for neonatal resuscitation such as RETAIN provide many opportunities for HCPs to acquire knowledge and practice their skills in a low-cost, low-stakes, and enjoyable learning environment that provides guidance and feedback, opportunities for repetition of missteps, and various levels of difficulty. They may help HCPs interpret complex situations better and thus complement routine refresher courses. This study adds to the literature that shows support for the integration of computer-based simulations in medical education by examining both noncognitive and cognitive factors, as well as the relationship between them.

Future work could also explore collaborative computer-based simulators of neonatal resuscitation. This study used a single-player game simulator, but extensions to multiplayer experiences could test the hypothesis that miscommunications among the team members decrease overall individual performance and infant outcomes.

### Conclusions

This study examined 50 HCPs’ performance as well as their mindsets and attitudes toward the terminology employed in the RETAIN computer-based simulator. The results revealed that the more the HCPs agreed that the terminology used in the tutorial and in the game did not impede their game experience, the better they performed in the game, but only when they endorsed more of a growth mindset. Conversely, the more they agreed with the terminology statements, the worse they performed in the game, but only when they endorsed more of a fixed mindset. These findings suggest that HCPs’ noncognitive factors such as the perception of the game and their mindsets have a significant impact on their actual performance in the game medium. Thus, one research direction could explore complex cognitive and noncognitive processes that may drive a set of HCP behaviors leading to better neonatal resuscitation performance. As computer-based game simulators are less costly and more practical than traditional training in neonatal resuscitation, future research will examine the role of computer-based game simulators in improving the safety of neonatal resuscitation procedures in the delivery room by investigating potential knowledge transfer, especially for high-risk deliveries.

## Appendix

Multimedia Appendix 1 Supplementary figures.

## Abbreviations

HCP: health care provider
NICU: neonatal intensive care unit
NRP: Neonatal Resuscitation Program
RETAIN: REsuscitation TrAINing
VIF: variance inflation factor
