# Supplementary material for: Health Care Providers’ Performance, Mindset, and Attitudes Toward a Neonatal Resuscitation Computer-Based Simulator: Empirical Study
Source: JMIR Serious Games. 2020 Dec 21;8(4):e21855. doi: 10.2196/21855 (PMC7781798; doi:10.2196/21855)
Supplement: Multimedia Appendix 1 [file games_v8i4e21855_app1.docx]

**Multimedia Appendix 1**

**Multiple Linear Regression Assumptions: Model 1**

**
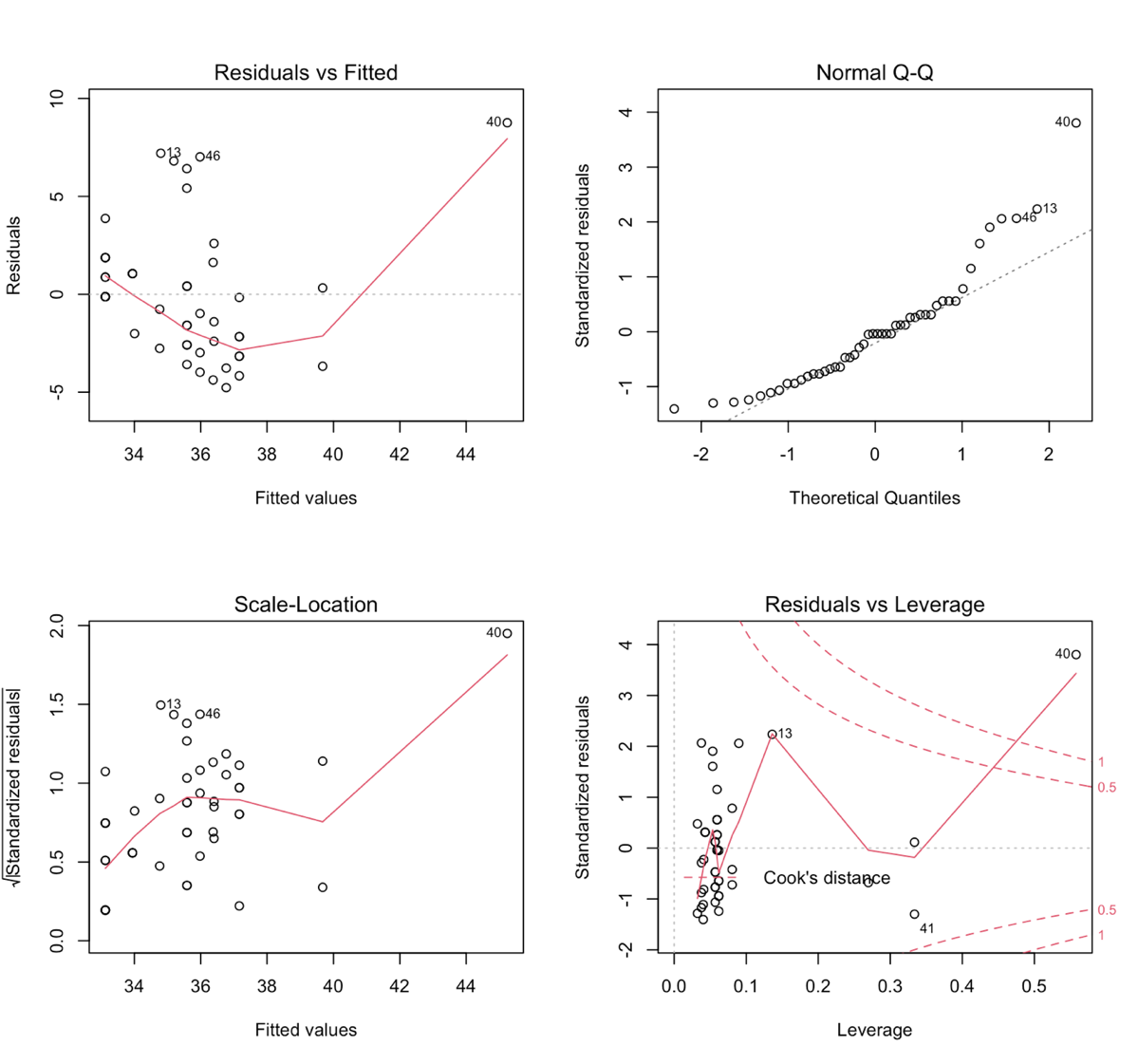
**

*Figure S1. Model 1 Linear Regression Assumptions*


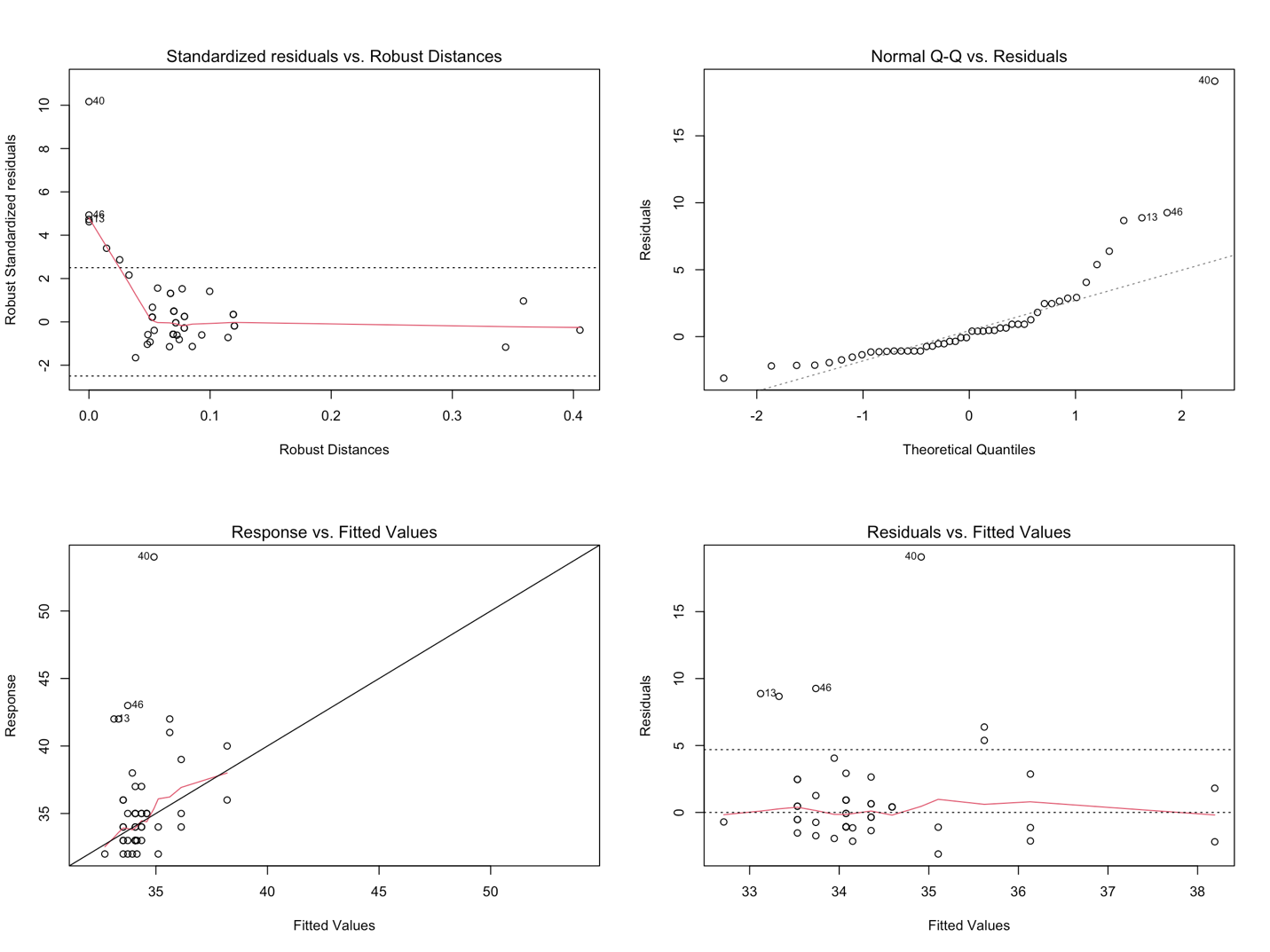


*Figure S2. Robust Model 1 Linear Regression Assumptions – Part 1*


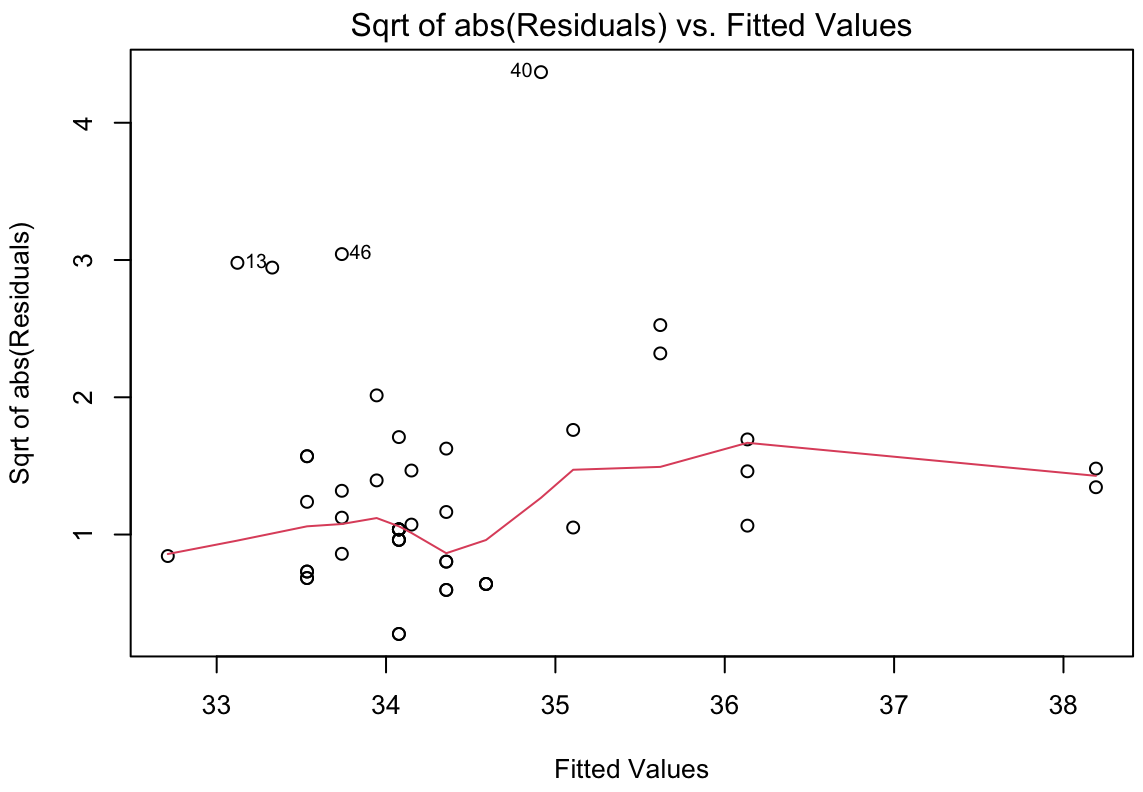


*Figure S3. Robust Model 1 Linear Regression Assumptions – Part 2*

**Multiple Linear Regression Assumptions: Model 2**

**
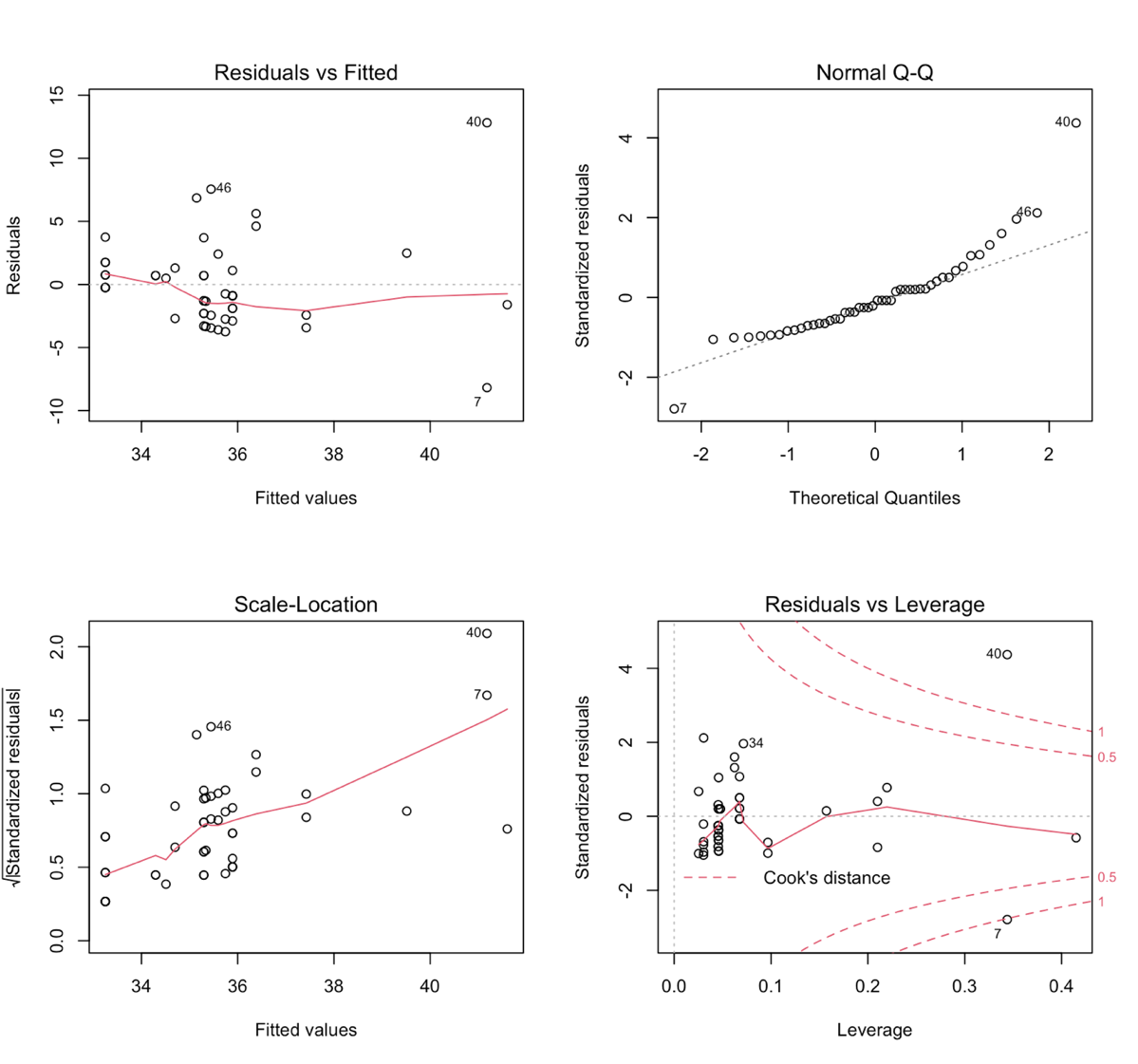
**

*Figure S4. Model 2 Linear Regression Assumptions*


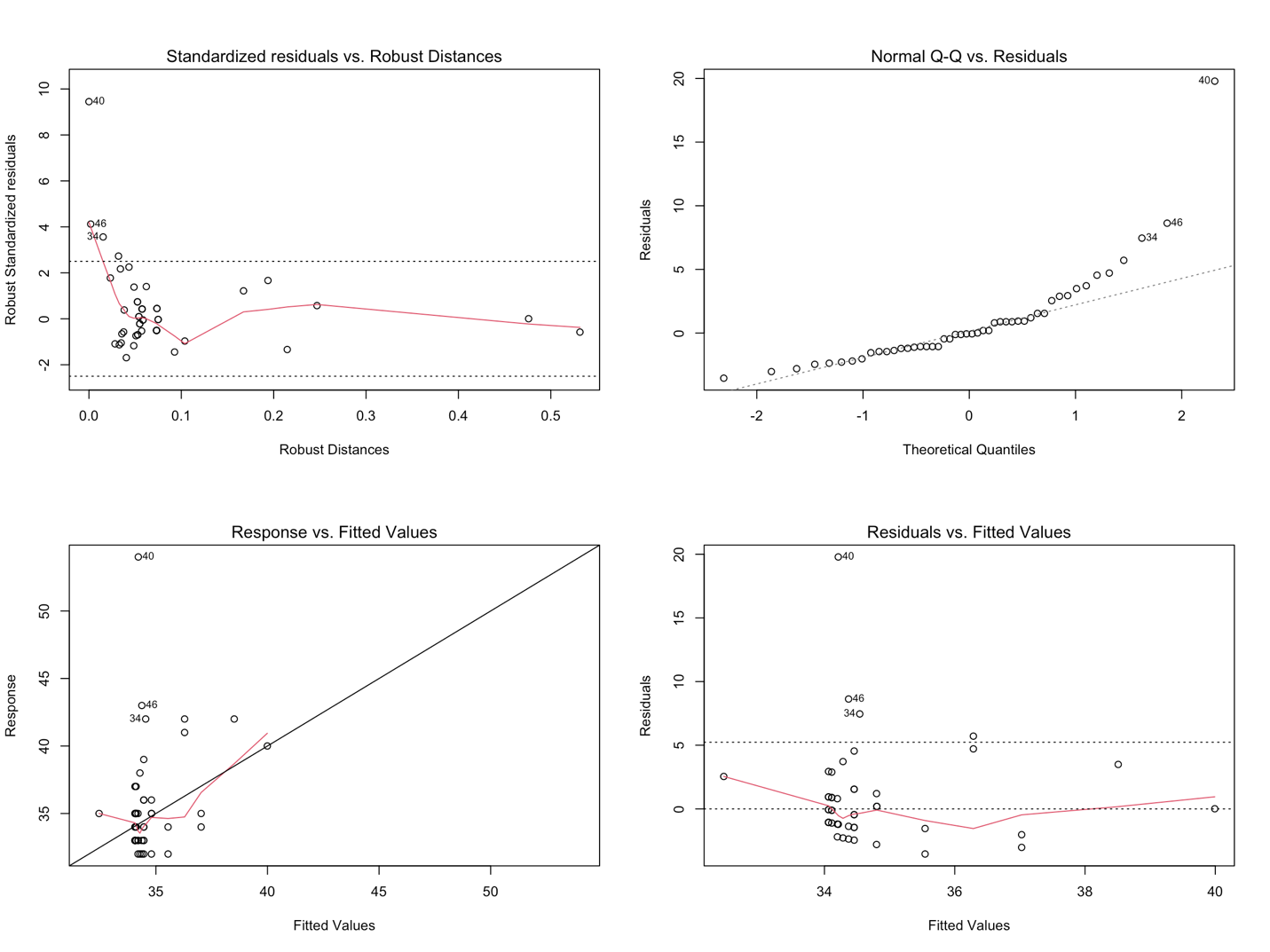


*Figure S5. Robust Model 2 Linear Regression Assumptions – Part 1*

**
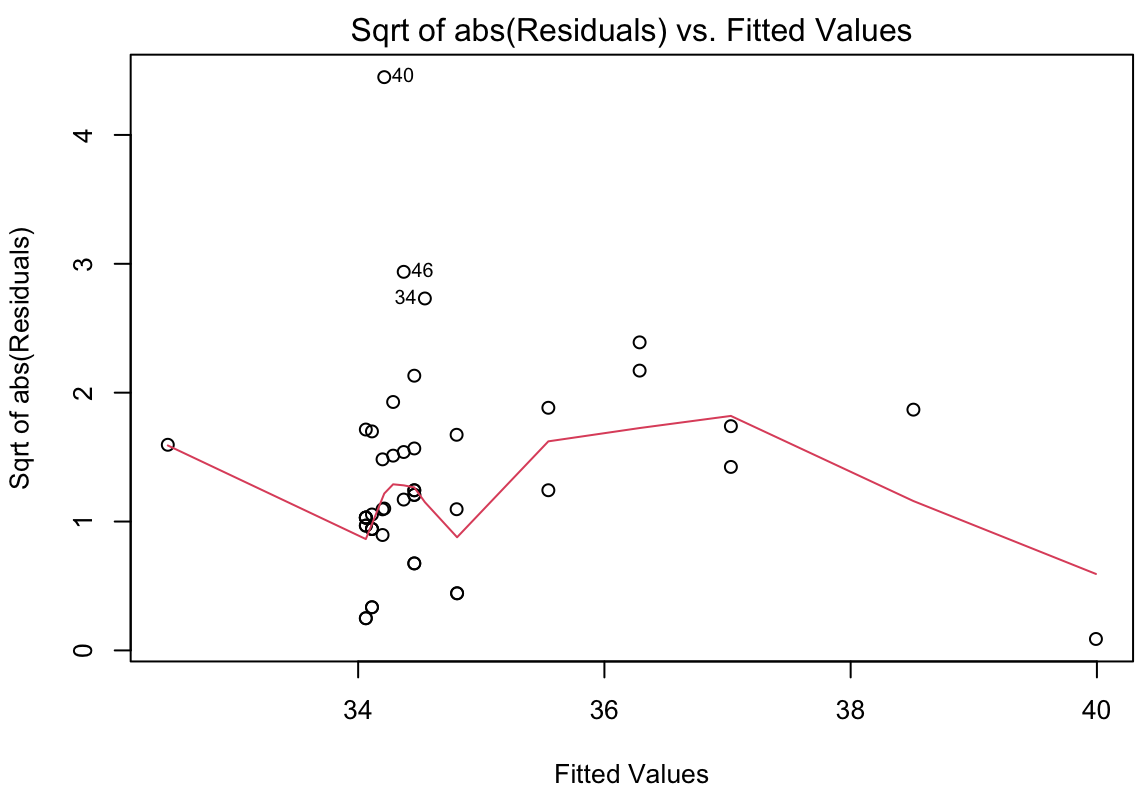
**

*Figure S6. Robust Model 2 Linear Regression Assumptions – Part 2*
